# Decreased 11β-Hydroxysteroid Dehydrogenase 1 Level and Activity in Murine Pancreatic Islets Caused by Insulin-Like Growth Factor I Overexpression

**DOI:** 10.1371/journal.pone.0136656

**Published:** 2015-08-25

**Authors:** Subrata Chowdhury, Larson Grimm, Ying Jia Kate Gong, Beixi Wang, Bing Li, Coimbatore B. Srikant, Zu-hua Gao, Jun-Li Liu

**Affiliations:** 1 Fraser Laboratories for Diabetes Research, Department of Medicine, the Research Institute of McGill University Health Centre, Montreal, Canada; 2 Department of Pathology, the Research Institute of McGill University Health Centre, Montreal, Canada; 3 Montreal Diabetes Research Centre, Montreal, Canada; Shanghai Jiaotong University School of Medicine, CHINA

## Abstract

We have reported a high expression of IGF-I in pancreatic islet β-cells of transgenic mice under the metallothionein promoter. cDNA microarray analysis of the islets revealed that the expression of 82 genes was significantly altered compared to wild-type mice. Of these, 11β-hydroxysteroid dehydrogenase 1 (11β-HSD1), which is responsible for the conversion of inert cortisone (11-dehydrocorticosterone, DHC in rodents) to active cortisol (corticosterone) in the liver and adipose tissues, has not been identified previously as an IGF-I target in pancreatic islets. We characterized the changes in its protein level, enzyme activity and glucose-stimulated insulin secretion. In freshly isolated islets, the level of 11β-HSD1 protein was significantly lower in MT-IGF mice. Using dual-labeled immunofluorescence, 11β-HSD1 was observed exclusively in glucagon-producing, islet α-cells but at a lower level in transgenic vs. wild-type animals. MT-IGF islets also exhibited reduced enzymatic activities. Dexamethasone (DEX) and DHC inhibited glucose-stimulated insulin secretion from freshly isolated islets of wild-type mice. In the islets of MT-IGF mice, 48-h pre-incubation of DEX caused a significant decrease in insulin release, while the effect of DHC was largely blunted consistent with diminished 11β-HSD1 activity. In order to establish the function of intracrine glucocorticoids, we overexpressed 11β-HSD1 cDNA in MIN6 insulinoma cells, which together with DHC caused apoptosis and a significant decrease in proliferation. Both effects were abolished with the treatment of an 11β-HSD1 inhibitor. Our results demonstrate an inhibitory effect of IGF-I on 11β-HSD1 expression and activity within the pancreatic islets, which may mediate part of the IGF-I effects on cell proliferation, survival and insulin secretion.

## Introduction

Insulin-like growth factor I (IGF-I) stimulates proliferation of pancreatic islet cells in a glucose-dependent manner, protects the β-cells against the development of diabetes mellitus, and exerts insulin-like effects on insulin target tissues [1, 2]. It activates IGF-I receptor through tyrosine phosphorylation and recruits intracellular substrates such as insulin receptor substrates (IRS) 1–4 and Shc, which trigger activations of PI3K/Akt, Ras/MAPK (Erk) and c-Jun N-terminal kinase (JNK) pathways [3], leading to stimulation on protein synthesis, cell survival and proliferation. We have previously reported that MT-IGF mice exhibit both highly concentrated IGF-I overexpression in the β-cells of the pancreas and significant resistance to streptozotocin-induced diabetes [4]. In order to explore novel molecular targets that mediate IGF-I actions, we recently performed a whole-genome cDNA microarray analysis on total RNA prepared from isolated islets of MT-IGF mice, and found 82 genes specifically up- or down-regulated [5]. HSD11B1, encoding 11β-hydroxysteroid dehydrogenase 1 (11β-HSD1), was one of the prominent targets previously not shown to be normally expressed in the pancreatic islets nor regulated by IGF-I.

It has been known that glucocorticoids at pharmacological concentrations directly affect β-cell integrity and function [1, 6, 7]. Either decreased or increased insulin secretion has been reported in response to both acute (in minutes) and prolonged (hours to days) exposure. However, the role of intracrine production of active glucocorticoids within the islets is poorly understood. As a reductase, 11β-HSD1 catalyzes the conversion of inert cortisone in humans (11-dehydrocorticosterone, DHC in rodents) to active cortisol (corticosterone) in the liver and adipose tissues, while the isozyme 11β-HSD2 (and 11β-HSD1 *in vitro*) catalyzes the reversed process of dehydrogenation (inactivating human cortisol into cortisone) [8]. Supporting its role in causing β-cell failure, the level of 11β-HSD1 was found to be increased in the islets of diabetic rodents [6, 9]. In order to establish 11β-HSD1 as a novel target of IGF-I action and its physiological function, we first confirmed the localization of 11β-HSD1 in pancreatic islets at the protein level, then characterized the changes in 11β-HSD1 level and activity caused by IGF-I overexpression and/or direct treatment, and assessed its functional relevance to glucose-stimulated insulin secretion (GSIS). Our results seem to support an IGF-I-induced inhibition on this enzyme and intracrine production of glucocorticoids, the latter of which is being developed as a molecular target in anti-obesity and anti-diabetic interventions [10, 11].

## Methods and Materials

### Transgenic mice, pancreatic islet isolation and insulin secretion

MT-IGF mice used in this study have been reported by us previously [4, 5]; the Research Institute Animal Care Committee of McGill University Health Centre (permit number: 2012–7052) approved this study and all animal handling including the sacrifice procedures. MT-IGF and wild-type mice were anesthetized using ketamine-xylazine-acepromazine at 50/5/1 mg/kg i.p. and sacrificed by cervical dislocation; pancreatic islets were isolated by collagenase digestion and allowed to recover for 3 h in DMEM medium containing 11 mM glucose and 10% fetal bovine serum, 10 mM HEPES, 2 mM glutamine, and 1 mM sodium pyruvate as reported [12]. They were hand-picked either for pooled RNA and protein isolations, or to test for acute insulin secretion by being divided into a 12-well plate (20 islets/well) and cultured overnight at 37°C in a humidified atmosphere containing 5% CO_2_. Where indicated, the islets were pre-treated with or without 100 nM dexamethasone (DEX) and DHC for 48 h. For GSIS, the islets were pre-incubated with low glucose (3.3 mM) in Krebs-Ringer bicarbonate buffer for 1 h, and switched to those containing 16.7 mM glucose for another hour. The supernatant and disrupted islets in lysis buffer (70% Ethanol, 0.18 N HCl) were stored at -80°C until required for insulin measurement using ELISA (Alpco, Salem, NH).

### Western blot analysis

Freshly isolated islets from 3–4 month old male mice were sonicated in 150–200 μl lysis buffer supplemented with protease inhibitor tablet (Roche Diagnostics, Basel, Switzerland). The cell extract was diluted by 1–1.5 volume Laemmli loading buffer containing 5% β-mercaptoethanol (Sigma-Aldrich, St. Louis, MO) and boiled for 5 min before loading onto SDS-PAGE gels. Together with liver and visceral fat extracts, Western blotting was performed to quantify protein levels of 11β-HSD1 (1:250, H-10 sc-20175, Santa Cruz, Dallas, TX) and β-actin (Santa Cruz or Medimabs, Montreal, QC). Similarly, MIN6 cells were used to probe for total and cleaved caspase-3 (1:1000, Cell Signaling, Danvers, MA).

### *In vitro* direct stimulation by IGF-I

Similar to our previous report [5], freshly isolated pancreatic islets pooled from 3 wild-type mice were allowed to recover overnight in culture medium containing 11 mM glucose and 10% fetal bovine serum [13]. In a 24-well plate, islets were distributed in triplicates of 20 for each condition and were cultured for 0 to 72 h in the same medium but contained only 1% fetal bovine serum upon treatment with or without 10^−8^ M of recombinant human IGF-I, Long R3 (I1271, Sigma-Aldrich). Total protein lysate was used for Western blots against 11β-HSD1 and β-actin. To study its effect on protein degradation, MIN6 cells overexpressing 11β-HSD1 (MIN6-HSD1, see below) were treated with cyclohexmide (CYC003, Bioshop; 10 mg/L, 2 h) to block protein synthesis before IGF-I treatment for 12–48 h.

### Dual-labeled immunofluorescence and immunohistochemistry

Paraffin sections of the pancreas taken from 3–5 month old, male MT-IGF and wild-type littermates were dewaxed, rehydrated, and blocked with 10% donkey serum, followed by incubation with rabbit anti-11β-HSD1 antibodies (1:100; H-10 sc-20175, Santa Cruz and ab83522, Abcam, Cambridge, MA) at 4°C overnight. After washing with PBS, sections were independently stained with anti-glucagon (C-18 sc-7779, Santa Cruz) and guinea pig polyclonal anti-insulin (ab7842, Abcam) followed by Alexa Fluor 594 conjugated donkey anti-rabbit IgG (H+L) and Alexa Fluor 488 goat anti-guinea pig IgG (Life technologies, Carlsbad, CA) [14, 15]. The images were analyzed using Axioshop 2 plus microscope (Carl Zeiss, Jena, Germany), Retiga 1300 digital camera, and Northern Eclipse software (Empix Imaging, Mississauga, ON). Paraffin sections of liver and pancreata taken from 3–5 month old male wild-type mice were incubated overnight with anti-11β-HSD1 (ab83522) with or without a specific blocking peptide (ab99223) at 4°C. After washing, the sections were incubated with secondary antibody and stained with diaminobenzidine substrate (Vector Laboratories). The microscopic images were analyzed using BX61 UIS2 Optical System microscope (Olympus) and Olympus stream software.

### Liver microsomal preparation

Microsomes were prepared from livers of male C57BL/6 mice after being sacrificed, homogenized in 0.5 ml/mg tissue TED buffer (10 mM Tricine, 1.5 mM EDTA, and 1 mM dithiothreitol, pH 7.4) using a glass homogenizer and a loose-fitting Teflon piston with intermittent cooling. The homogenate was first cleared by being micro centrifuged at 13,000 g for 30 min at 4°C, and the supernatant was further centrifuged at 105,000 g for 60 min at 4°C. The pellet was washed in TED buffer, centrifuged again at 105,000 g for 30 min at 4°C, and resuspended in TED buffer [9]. The protein concentration was determined by the Bradford method using Bio-Rad protein assay.

### *In vitro* dehydrogenase and reductase assays of 11β-HSD1 activity

11β-HSD1 is a bidirectional enzyme with both reductase and dehydrogenase activities [16]. To measure dehydrogenase activity, the conversion of corticosterone to DHC was measured by incubating 150 freshly isolated islets from each mouse for 24 h with 1.5 nM [1,2,6,7-^3^H]-corticosterone (Perkin-Elmer, Waltham, MA). The steroids were extracted from the medium with ethyl acetate by vigorously vortexing and being centrifuged at 2,000 g for 10 min at 4°C. The upper phase extract was evaporated under nitrogen. The steroids were then dissolved in 450 μl methanol, spotted on a thin layer chromatography (TLC) plate with unlabeled DHC and corticosterone as reference markers, and resolved with chloroform-methanol (95:5 v/v). Liver microsomes were also used as a positive control in comparison to the changes in pancreatic islets. TLC plates were first dried and placed in an enclosure containing iodine resublimed crystals (Fisher Scientific, Toronto, ON). Yellow-brownish spots appearing in regions containing DHC and corticosterone were scraped off, eluted with ethyl acetate, dried under nitrogen and mixed with LSC-cocktail (ScientiSafe Gel, Fisher). The fractional conversion of corticosterone to DHC was calculated by analyzing LSC-cocktail mixture in liquid scintillation analyzer Ti-Carb 2801TR (Perkin Elmer). The dehydrogenase activity was expressed as percentage radioactivity of the fractions [17].

Alternatively, the established *in vivo* role of 11β-HSD1 is reductase-directed conversion of inert DHC into corticosterone in rodents, the activity of which was measured by the conversion of human cortisone to cortisol from freshly isolated islets and liver microsome. Batches of 300 islets from each mouse were incubated with 500 nM cortisone (Sigma-Aldrich) for 3 h. The steroids were extracted from the medium and the cortisol concentration was measured using ELISA (Enzo Life Science, Farmingdale, NY) as reported [18].

### Stable overexpression of 11β-HSD1 cDNA and proliferation assay in MIN6 cells

To directly assess the function of 11β-HSD1 *in vitro*, mouse cDNA with a 3'-(HA)_3_ tag was sub-cloned into pcDNA3.1 vector (Invitrogen, Thermo Fisher Scientific, Waltham, MA) between the cytomegalovirus promoter and bovine growth hormone polyadenylation sequence and used to transfect murine insulinoma MIN6 cells, which were selected using G418 (Wisent, St-Bruno, QC) for 60 d as reported [5]. After Western blot confirmation using anti-HA tag antibody (Cat# G036 Abm, Richmond, BC), 11β-HSD1-overexpressing (MIN6-HSD1) and vector transfected (MIN6-Vec) clones were subjected to 3-(4,5-dimethylthiazol-2-yl)-2,5-diphenyltetrazolium (MTT) cell viability (Sigma-Aldrich) and 5-bromo-2'-deoxyuridine (BrdU) incorporation assays. The cells were cultured in 10% serum for 1 to 3 d in the presence or absence of 11β-HSD1 inhibitor 10j (Cat# 385581, Calbiochem, EMD Millipore, Etobicoke, ON) before the assays were performed. For BrdU incorporation, in the final 18 h of incubation, 10 μM BrdU was added; its incorporation was quantified using ELISA at 450 nm (EMD Millipore).

### Dehydrocorticosterone (DHC)-induced apoptosis in MIN6-HSD1 cells

MIN6-HSD1 cells were sub-cultured at a density of 2 x10^4^ cells/cm^2^ and incubated with or without 100 nM DHC and 1 μM of 11β-HSD1 inhibitor for 72 h. DEX (100 nM) was added in additional wells as a positive control to cause apoptosis. The occurrence of apoptosis was represented by the amount of DHC/DEX-induced caspase-3 activation which is quantified by Western blot using cleaved caspase-3 antibody (9661, Cell Signaling). In separate experiments, the change of cleaved caspase-3 level was also determined using immunofluorescence staining in MIN6-HSD1 cells treated with DHC alone, DHC and 11β-HSD1 inhibitor, or vehicle only. 4% Paraformaldehyde fixed cells were permeabilized with 0.1% Triton X-100 in TBS for 10 min, probed with DAPI (blue) and cleaved caspase-3 antibody followed by Alexa 488 conjugated goat anti-rabbit IgG (green). Images were taken on an Axioskop 2 Plus microscope (Carl Zeiss) at 200X magnification.

The presence of mono- and oligonucleosomes in apoptosis were measured by histone-associated DNA fragments in the cytoplasm, using sandwich enzyme immunoassay cell death detection ELISA plus kit (Roche, Cat#11774425001) [19]. Both MIN6-HSD1 and MIN6-Vec cells, after being treated for 72 h with or without DHC and 11β-HSD1 inhibitor, were collected by being centrifuged at 200 g for 10 min and lysed for 30 min with the buffer provided. The cell lysate was cleared by centrifugation again at 200 g for 10 min. Aliquots of the supernatant (20 μl, representing the cytosolic fraction) were transferred to streptavidin-coated wells and incubated with anti-histone-biotin and anti-DNA-peroxidase antibodies for 2 h, followed by the 2,2'-azino-bis(3-ethylbenzthiazoline-6-sulfonic acid) substrate for 10 min, and measured as a ratio of absorbance at 405 and 490 nm using Perkin Elmer Enspire multiplate reader.

### Statistical analysis

Data were expressed as Mean ± S.E. and plotted using Sigma Plot version 11 (Systat Software, San Jose, CA), which was also used to perform ANOVA and pot-hoc Holm-Sidak test. Unpaired Student’s t-test was performed using GraphPad InStat version 3. *P* values <0.05 were considered to be significant.

## Results

### Decreased 11β-HSD1 protein level in the islets of IGF-I overexpressing mice

In order to identify novel targets of IGF-I action, we recently performed whole-genome cDNA microarray analysis on freshly isolated pancreatic islets of MT-IGF and wild-type mice. Amongst those was CCN5/WISP2 which was functionally evaluated and reported by us [5]. In this study, we report the elucidation of 11β-HSD1, another IGF-I regulated target which catalyzes the intracellular conversion and activation of glucocorticoids as previously reported in the liver, skeletal muscles and adipose tissues but not in pancreatic islets [20].

**Fig 1:**
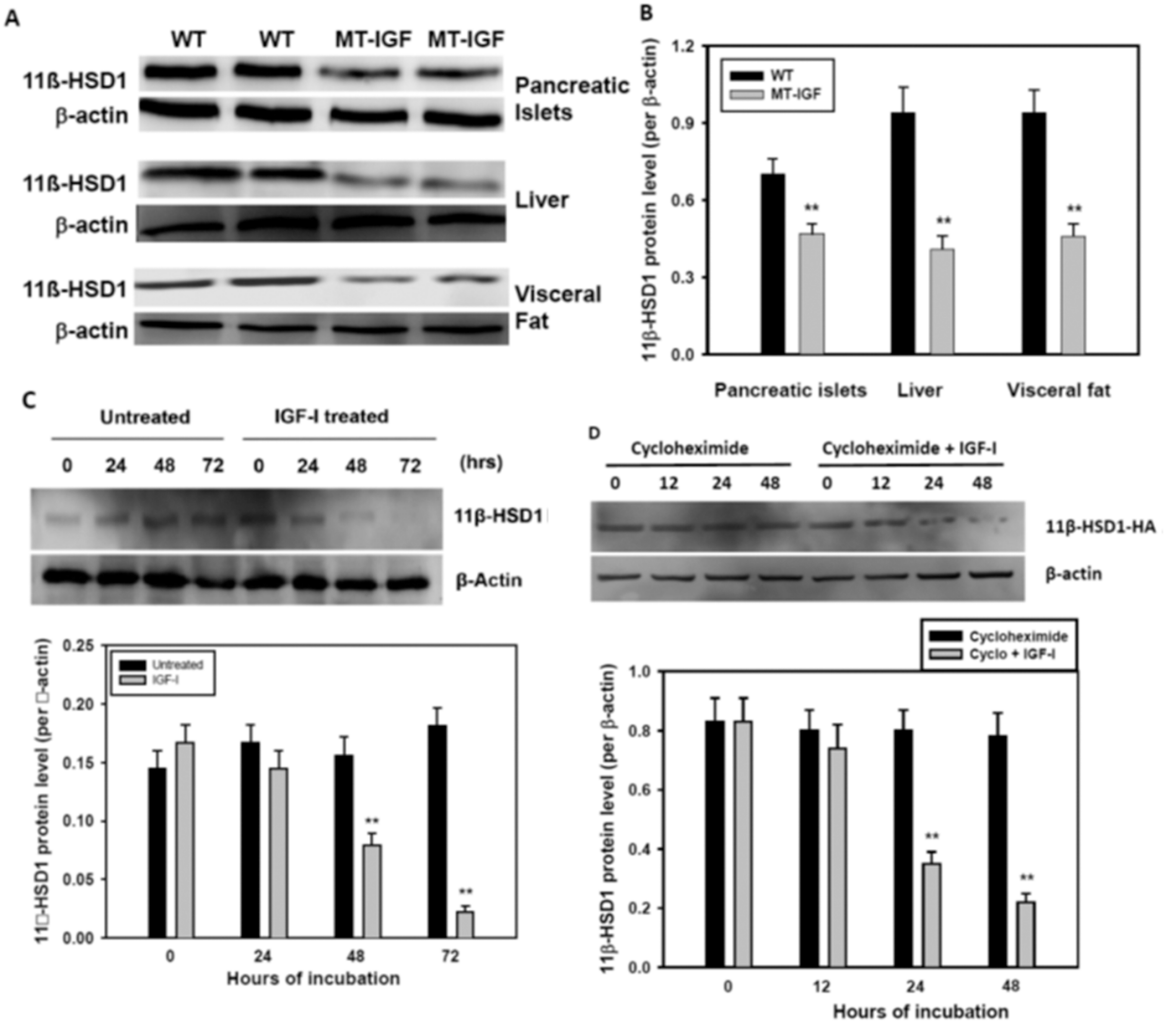
Decreased 11ß-HSD1 protein level by IGF-I overexpression *in vivo* and a direct treatment with IGF-I. **A**. IGF-I overexpression decreased the levels of 11ß-HSD1 in pancreatic islets, liver and visceral fat, shown in Western blots. Wild-type and MT-IGF littermates, 3–4 months old, were sacrificed to isolate pancreatic islets, liver and visceral fat; cell lysates were loaded on blots which were probed against 11β-HSD1 and β-actin. **B**. The result of Western blot densitometry analysis. Mean ± SE, N = 6, **P<0.01 vs. wild-type islets. **C**. Decreased 11β-HSD1 protein level caused by direct IGF-I treatment in freshly isolated islets from wild-type mice. Long R3 IGF-I, Sigma-Aldrich, 10 nM or vehicle was added; the islets cells were harvested after 1, 2, or 3 d. The result of Western blotting was corrected with that of β-actin. N = 4, **P<0.01 vs. untreated. **D**. IGF-I treatment accelerated 11β-HSD1 protein degradation in 11β-HSD1 overexpressing MIN6 cells (as in Figs 5 and 6). MIN6-HSD1 cells were treated with cycloheximide (10 mg/L) for 2 h before IGF-I (10 nM) or vehicle for 12–48 h. N = 4, **P<0.01 vs. cyclohexmide alone.

To further establish functional relevance of the microarray screening, we compared the levels of 11β-HSD1 protein in freshly isolated islets from wild-type and MT-IGF mice. Although the microarray screening and qRT-PCR confirmation showed a significant *increase* in 11β-HSD1 mRNA level (S1 Fig) [5], we observed a 35% *decrease* in the protein level in the islets of MT-IGF vs. wild-type littermates (Fig 1A and 1B). The finding of 11β-HSD1 regulation by IGF-I in the islets is novel, although the inhibitory effect has been established in the liver and adipose tissues [8, 21]; the latter was clearly confirmed in Fig 1A and 1B. In order to determine if these findings arise from direct or indirect effects of IGF-I, we treated freshly isolated wild-type islets with 10 nM IGF-I for 1–3 d, which revealed a late-onset inhibition on 11β-HSD1 protein level (Fig 1C). At 48 and 72 h, the protein level was significantly decreased to 51% and 12% of untreated islets supporting a direct inhibition by IGF-I. To further pinpoint possible effects on protein degradation, we blocked protein synthesis in 11β-HSD1-overexpressing MIN6 cells (see below, under “Decreased rate of cell proliferation caused by 11β-HSD1 overexpression”) and still observed a clear reduction in 11β-HSD1 protein level after 24 and 48 h of IGF-I treatment, suggesting that IGF-I accelerates its degradation.

### Islet α-cell specific 11β-HSD1 expression and its reduction by IGF-I overexpression

**Fig 2:**
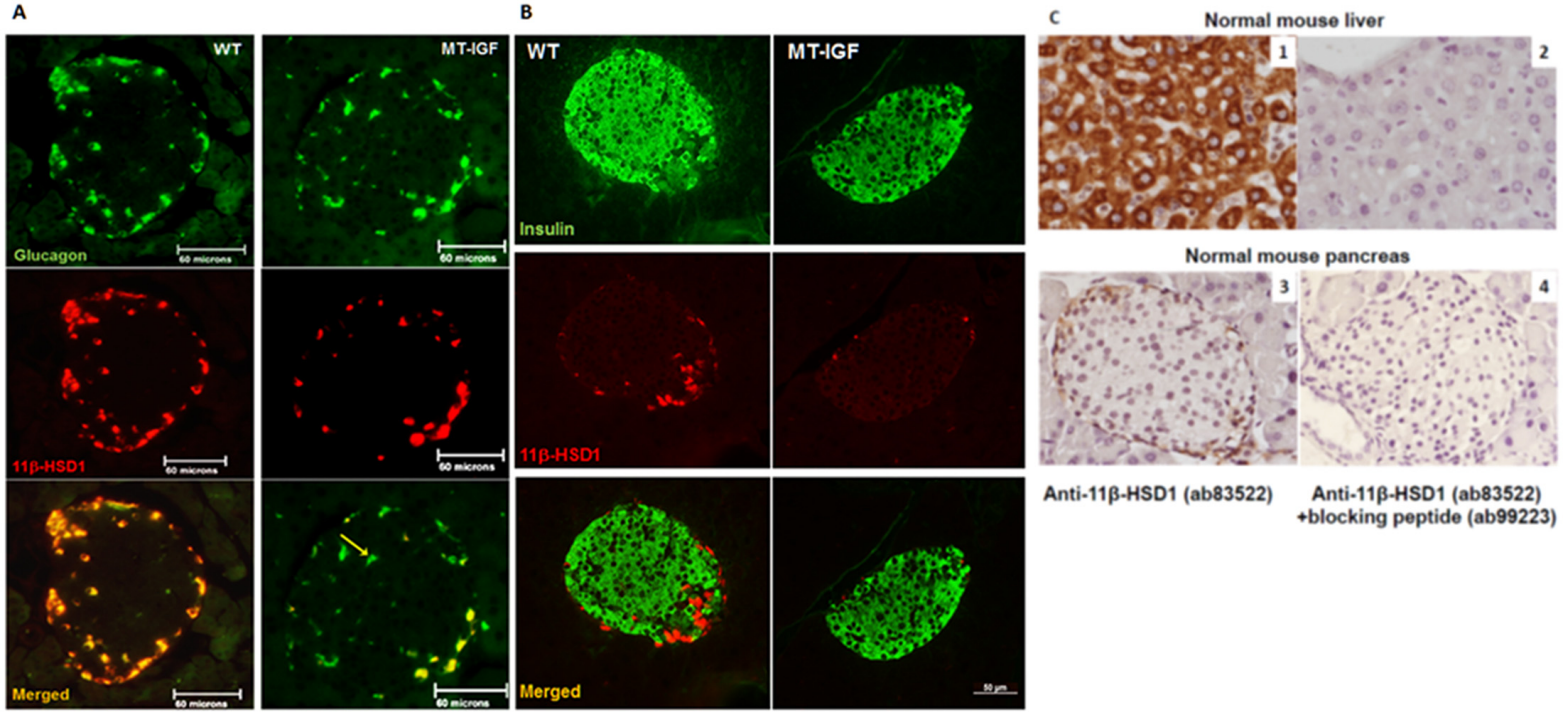
Pancreatic islet α-cell-specific expression of 11β-HSD1 and its inhibition by IGF-I overexpression. **A**. Paraffin sections of the pancreas taken from 3-month-old wild-type (WT) or MT-IGF mice were stained for glucagon using DY488- (upper panels) and 11β-HSD1 using rhodamine-conjugated secondary antibodies (middle panels). Consequently, the images were merged into the bottom panels using Northern Eclipse software. 11β-HSD1 staining was distributed in the cytosol of most α-cells (68% on average) but not in other endocrine or acinar cells in wild-type mice (left panels); diminished staining per cell, some totally devoid of 11β-HSD1 (yellow arrow), was revealed in MT-IGF mice (right panels, only 21% of α-cells were positive for 11β-HSD1). Representative islets of ten from each genotype were illustrated. The scale bar was 60 microns. **B**. The same sections were stained for insulin using DY488- (upper panels) and 11β-HSD1 using rhodamine-conjugated secondary antibodies (middle panels). The scale bar was 50 microns. **C**. Test of antibody specificity using blocking peptide. Paraffin sections of normal mouse liver and pancreas were stained for 11β-HSD1 using ab83522 and DAB reagent (panels 1 and 3); as negative controls, the blocking peptide (ab99223) used in panels 2 and 4 abolished all specific staining. Images were taken at 400X magnifications.

To determine the localization of 11β-HSD1 in specific cell types within the islets, we performed dual-labeled immunofluorescence in reference to both glucagon- and insulin-producing α- and β-cells respectively (Fig 2A and 2B). In wild-type islets, 11β-HSD1 was only detected in islet α-cells, and in the majority of them, as has been reported [17] and further supported by a recent finding that isolated mouse islets express 11.7-fold higher level of 11β-HSD1 mRNA than purified β-cells from other endocrine cells using flow cytometry (S2 Table) [22]. They are in contrast to two other reports of β-cell expression, however [18, 23]. Islets from MT-IGF mice showed a distinct reduction in the protein level per cell with no change in α-cells localization. Besides, some islet α-cells become devoid of 11β-HSD1 (yellow arrow, Fig 2A). As a positive control for antibody specificity, we confirmed high level cytoplasmic staining of 11β-HSD1 in hepatocytes using two independent antibodies (Abcam and Santa Cruz). To further assess specificity, a specific blocking peptide (ab99223) abolished all cytoplasmic staining of 11β-HSD1 in mouse liver and pancreas (Fig 2C).

### Decreased 11β-HSD1 reductase and dehydrogenase activities in the islets of MT-IGF mice

**Fig 3:**
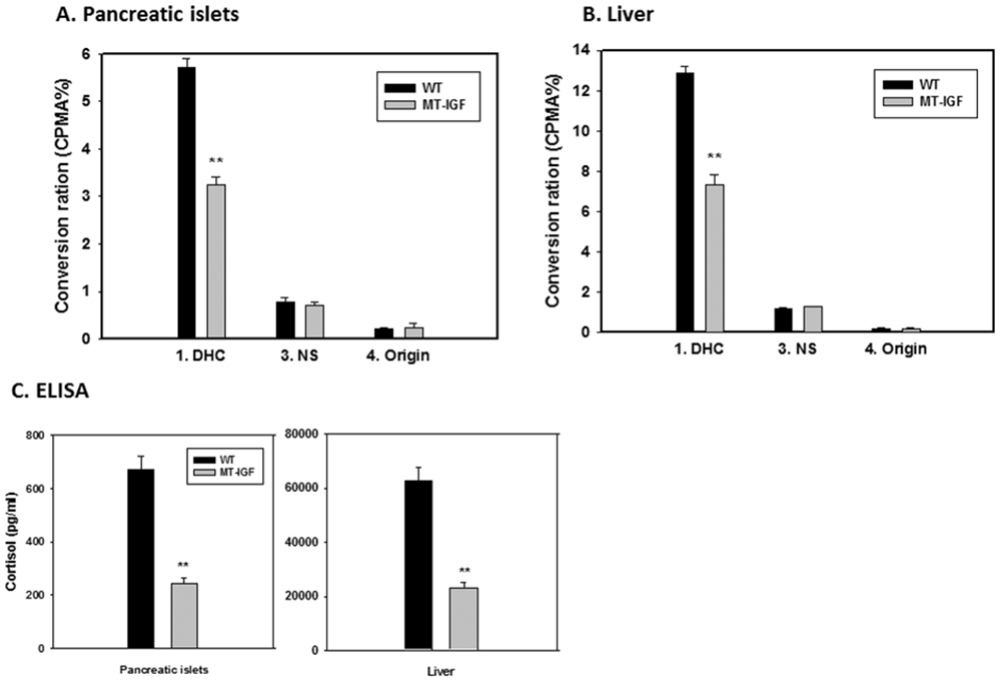
Decreased 11β-HSD1 activity in the liver and pancreatic islets of MT-IGF mice. **A**. Changes in DHC conversion rate in pancreatic islets. Radioactivity corresponding to [1,2,6,7-^3^H] DHC was expressed as percentage of the total radioactivity pooled from all four bands. Also illustrated were the radioactivities of the non-specific band (3. NS) and those remained at the loading spots (4. Origin). **B**. Changes in DHC conversion rate in the liver. Mean ± SE. N = 3. **P<0.01 vs. wild-type islets or liver. A representative assay was illustrated from three experiments. **C**. Decreased conversion of human cortisone to cortisol in the islets and liver of MT-IGF mice. Freshly isolated islets and liver microsomes from wild-type and MT-IGF mice were incubated with 500 nM cortisone for 3 h. Steroids were extracted and the concentration of newly converted cortisol was measured using the enzyme immunoassay. N = 3; **P<0.01.

Following the demonstrations *in vivo* using immunohistochemistry, Western blotting on isolated islets, and *in vitro* IGF-I treatment, we sought to further validate the decreased enzymatic activity. The notion 11β-HSD1 was not present in the islet β-cells but mainly expressed by the α-cells also requires further investigation to confirm its functional relevance. While normally being an NADPH-dependent reductase, 11β-HSD1 can also act as an NAD-dependent dehydrogenase *in vitro*, thus catalyzing the interchangeable conversion of DHC and corticosterone in rodents. We first isolated fresh islets from MT-IGF and wild-type mice and performed an *in vitro* dehydrogenase assay on the conversion of [1,2,6,7-^3^H]-corticosterone to DHC. The separation of different molecular forms was achieved by thin layer chromatography (TLC) and the radioactivity measured by liquid scintillation. On the TLC plates, in addition to the radioactive substrate and conversion product, we detected a third nonspecific intermediate which had very little radioactivity and no significant variation among MT-IGF and wild-type mice, in both the liver and pancreatic islet samples (data not shown). There was a significant 44% decrease in the rate of corticosterone dehydrogenation in the islets of MT-IGF vs. wild-type littermates (Fig 3A, first two bars). This reduction was consistent with the decrease of 11β-HSD1 protein level (Fig 1) and immunohistochemistry (Fig 2). We further confirmed a 45% decrease in 11β-HSD1 activity in liver microsomes of MT-IGF vs. wild-type mice (Fig 3B, first two bars) [24].

Alternatively, the established *in vivo* role of 11β-HSD1 in rodents is reductase-directed conversion of inert DHC into corticosterone, the activity of which was measured by the production of (human) cortisol from cortisone in isolated islets of wild-type and MT-IGF mice (Fig 3C, left panel). In the islets of MT-IGF mice it demonstrated a more significant 64% reduction. Together with the 44% decrease in dehydrogenase activity (Fig 3A), they support a net reduction in 11β-HSD1 protein level. The decrease in liver 11β-HSD1 reductase activity was also confirmed (Fig 3C, right panel).

### Differential effects of DEX and DHC on glucose-stimulated insulin secretion

**Fig 4:**
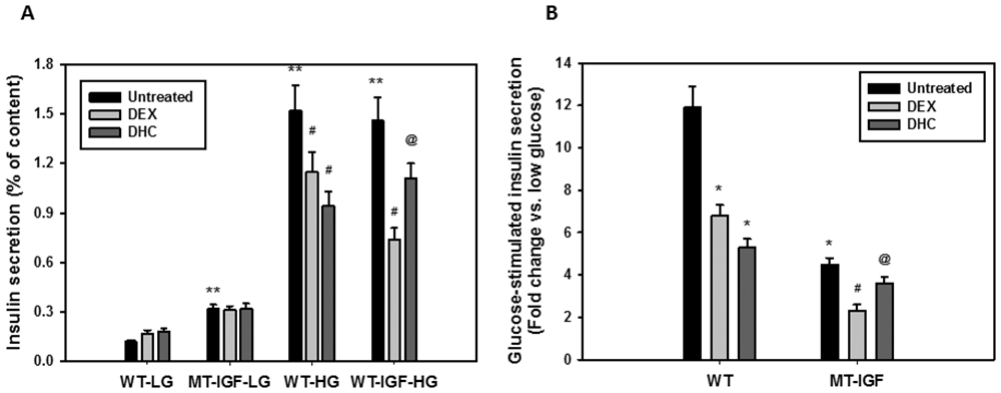
Effects of dexamethasone (DEX) and 11-dehydrocorticosterone (DHC) pre-incubations on glucose-stimulated insulin secretion. Freshly isolated islets from wild-type mice were pre-incubated in regular medium with 100 nM DEX or DHC for 48 h, first kept at 3.3 mM glucose (LG) for 60 min before being stimulated with 16.7 mM glucose (HG) for another 60 min. Insulin concentrations in the incubation buffer were measured using ELISA and expressed as % insulin content directly (A) or as fold stimulation vs. untreated (low glucose) islets (B). The experiment was repeated three times; a representative assay was illustrated. Mean ± S.E. N = 3. Results of 1-way ANOVA: (A) among the first 6 columns of 3.3 mM glucose, P<0.01; among the other 6 columns of 16.7 mM glucose, P<0.001. (B) Among all 6 columns P<0.001. *P<0.05, **P<0.01 vs. untreated wild-type islets; #P<0.05 vs. untreated MT-IGF islets; @P<0.05 vs. DEX-treated MT-IGF islets.

Although excess glucocorticoids inhibit insulin secretion and cause β-cell death [25], the role of locally generated glucocorticoids within the islets catalyzed by the action of 11β-HSD1 has not been established. We speculate that significantly decreased 11β-HSD1 activity in the pancreatic islets and the resulting decrease in intracrine glucocorticoid production constitute part of the IGF-I activity. In order to examine the effect on GSIS, we pre-incubated the islets for 48 h with 100 nM DEX or DHC, the latter requiring 11β-HSD1-mediated intracrine activation. As shown in Fig 4A, our islets exhibited a robust response in insulin release upon high glucose stimulation; MT-IGF islets seemed to have an elevated rate of basal release (4^th^ vs. 1^st^ bars). Both DEX and DHC caused significant 25–39% decreases in GSIS from wild-type islets (compare bars 8 and 9 vs. 7). In MT-IGF islets, DEX had an even stronger inhibition on GSIS (50% inhibition of 11^th^ vs. 10^th^ bars); however, the effect of DHC was significantly weakened (only 26% inhibition of 12^th^ vs. 10^th^ bars). We suggest the latter effect was related to the diminished 11β-HSD1 level and intra-islet conversion of glucocorticoids. The relative fold changes were illustrated in Fig 4B, which showed a significant decrease from 11.8 fold in wild-type islets to 4.5 fold in MT-IGF islets due in part to elevated basal insulin release. Although DEX still inhibited GSIS (from 4.5 to 2.3 fold), the effect of DHC was largely blunted (from 4.5 to 3.6 fold; bars 5 and 6 vs. 4), which seems to suggest that IGF-I overexpression causes diminished 11β-HSD1 level, decreases the activation of DHC in the islet cells, and partially rescues the inhibition of GSIS by intracrine glucocorticoids.

### Decreased rate of cell proliferation caused by 11β-HSD1 overexpression

**Fig 5:**
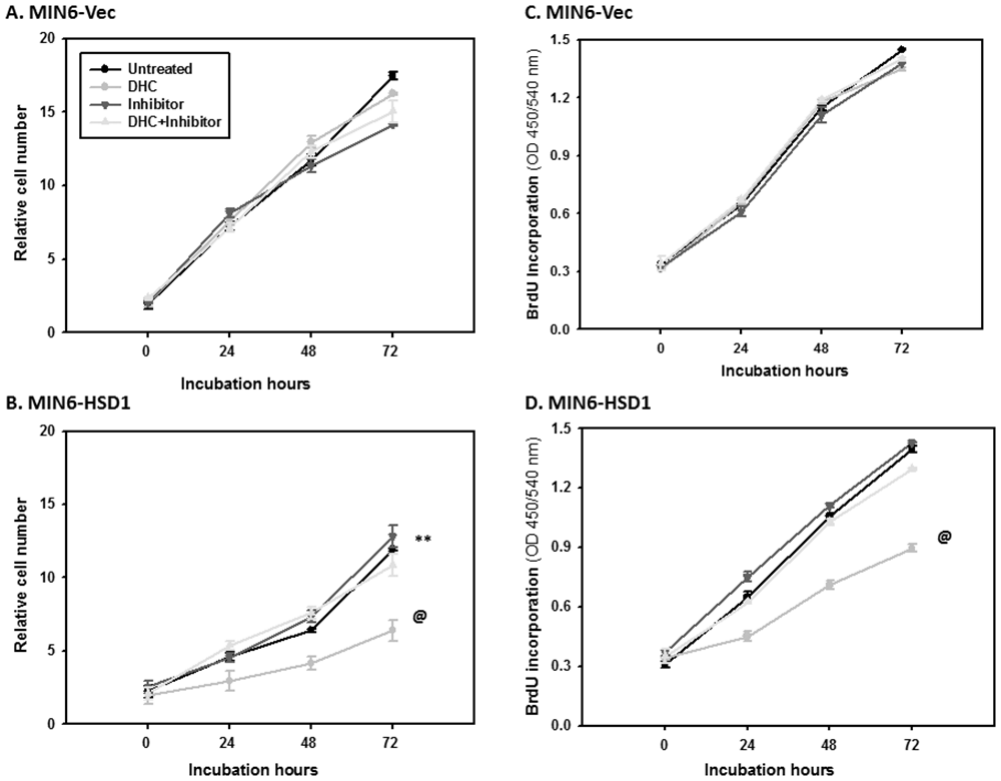
Decreased cell proliferation in MIN6 cells overexpressing 11β-HSD1 in the presence of DHC. In A and B, MIN6-Vec and MIN6-HSD1 cells were cultured for 3 d with or without DHC (100 nM) and the 11β-HSD1 inhibitor (1 μM). Relative cell numbers were determined using the MTT assay. N = 10, **P<0.01 in untreated MIN6-HSD1 vs. MIN6-Vec cells using one-way ANOVA. @P<0.01 in DHC-treated vs. untreated MIN6-HSD1 cells using one-way ANOVA. In C and D, cells were cultured for 3 d with or without DHC and the 11β-HSD1 inhibitor. The relative rate of cell proliferation was determined using BrdU incorporation into newly synthesized DNA. N = 10, @P<0.01 in DHC-treated vs. untreated MIN6-HSD1 cells using one-way ANOVA.

To further define the effect of increased 11β-HSD1 level in islet function, we transfected its cDNA into MIN6 insulinoma cells using pcDNA3.1 vector and generated three independent, stable lines and confirmed significant overexpression of 11β-HSD1 protein in MIN6-HSD1 cells through Western blot (data not shown). Immediately we noticed that MIN6-HSD1 cells were somehow unhealthy and grew slower compared to MIN6-Vec which prompted us to assess cell viability using MTT. As shown in Fig 5A, MIN6-Vec cells with only low and endogenous 11β-HSD1 expression grew normally from 0 to 3 d, and relative cell numbers increased 11-fold. The addition of DHC did not cause much change, consistent with the fact that the cells only have low levels of endogenous 11β-HSD1. Consequently, 11β-HSD1 inhibitor had no effect on cells with or without DHC. However, MIN6-HSD1 cells grew much slower, and reached only a 7-fold increase in 3 d [vs. MIN6-Vec cells; ANOVA P<0.01] (Fig 5B). The addition of DHC further delayed cell number acceleration to only 2-fold; which was completely rescued by the 11β-HSD1 inhibitor (back to 7-fold). Thus, simple overexpression of 11β-HSD1 in MIN6 cells (with inert substrate DHC normally available in culture medium) either caused cell death or slowed proliferation, while increased DHC further deteriorated the situation. The 11β-HSD1 inhibitor was sufficient to rescue the effect of supplemented DHC but not enough to rescue all the negative effect caused by 11β-HSD1 overexpression.

To confirm decreased proliferation caused by 11β-HSD1 overexpression and/or added substrate DHC, we assessed BrdU incorporation. As shown in Fig 5C, there was a 4.8-fold increase in BrdU incorporation in MIN6-Vec cells after 3 d in culture. Addition of DHC and/or 11β-HSD1 inhibitor had no effect, supporting again that endogenous 11β-HSD1 level was low in these cells. In Fig 5D, overexpression of 11β-HSD1 itself had no effect on the proliferation of MIN6-HSD1 cells (the same 4.8-fold). Supplementation of DHC decreased BrdU incorporation from 4.8 to 3-fold (ANOVA P<0.01 between the two curves), an effect mostly rescued by the further addition of 11β-HSD1 inhibitor; however the inhibitor itself had no effect. Clearly in the presence of supplemented DHC, 11β-HSD1 overexpression inhibited cell proliferation but it cannot explain the entire decrease revealed using MTT assay (Fig 5A and 5B), which called for a study on 11β-HSD1 and intracrine glucocorticoid mediated cell death.

### 11β-HSD1 overexpression conferred DHC-induced apoptosis in insulinoma cells

**Fig 6:**
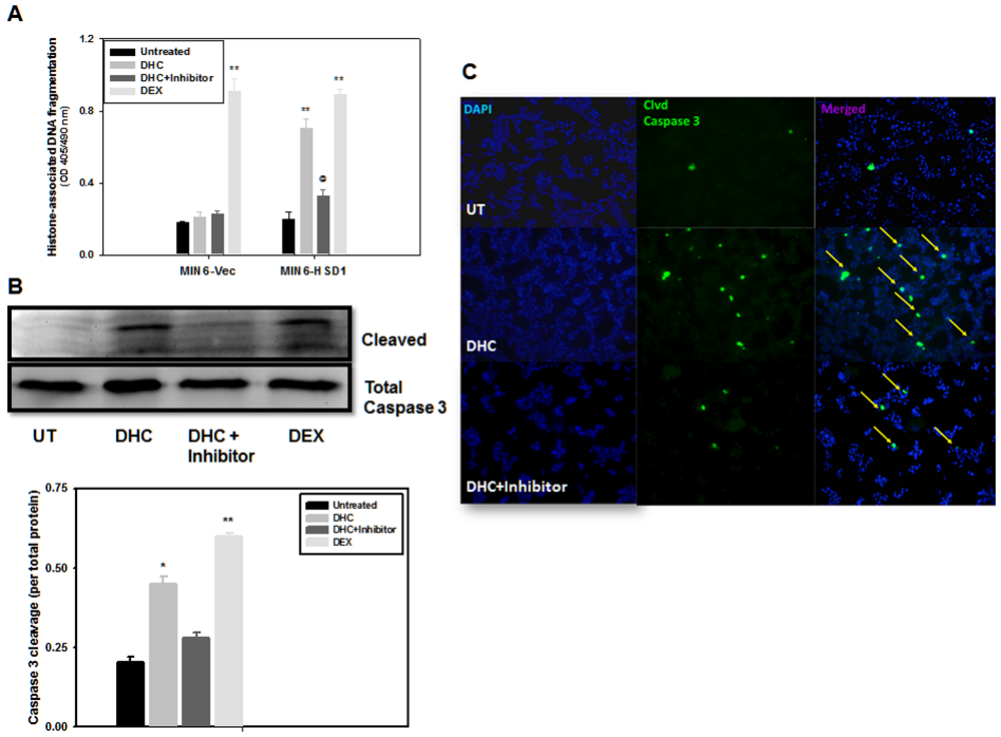
11β-HSD1 dependent, DHC-induced apoptosis in MIN6 cells. **A**. MIN6-Vec and MIN6-HSD1 cells were cultured for 3 d with or without DHC (100 nM) and the 11β-HSD1 inhibitor (1 μM). The relative rate of cell apoptosis was measured by cytoplasmic histone-associated DNA fragmentation using ELISA. N = 5, **P<0.01 vs. untreated MIN6 cells; @P<0.05 vs. DHC-treated MIN6-HSD1 cells. **B**. In a separate experiment but under the same condition as in A, caspase 3 cleavage in MIN6-HSD1 cells alone was quantified using Western blots and densitometry analysis was performed. N = 5, *P<0.05, **P<0.01 vs. untreated cells. **C**. In another experiment under the same condition as in A and B, MIN6-HSD1 cells were cultured in chamber slides, treated, and representative fluorescence images of cleaved caspase 3 were illustrated. Arrows indicate active caspase 3 cleavage which was diminished upon treatment with the11β-HSD1 inhibitor. Representative fields were illustrated from N = 5.

Glucocorticoids are known to induce cell apoptosis by directly regulating typical apoptotic or survival genes, or by inducing cellular distress that triggers the apoptotic cascade [26]. We investigated the effect of 11β-HSD1 overexpression and DHC incubation on cell death in stably transfected MIN6 lines, first measured by histone associated DNA fragmentation in the cytoplasm [5]. As shown in Fig 6A, in the first four bars, DEX treatment (4^th^ vs. 1^st^) in MIN6-Vec cells caused more than a 5.1-fold increase in the amount of DNA fragmentation; under the same condition, DHC (2^nd^ vs. 1^st^) had no effect, because of low endogenous 11β-HSD1 in MIN6 cells (undetected by Western blot; data not shown) to convert and activate it. In MIN6-HSD1 cells, both DHC and DEX caused 3.4- and 4.4-fold increases in DNA fragmentation respectively (6^th^ and 8^th^ vs. 5^th^ bars). Addition of the 11β-HSD1 inhibitor largely abolished the effect of DHC (7^th^ vs. 6^th^ bars), supporting the notion that 11β-HSD1 mediated DHC activation causes apoptotic cell death.

Caspase-3 activation and cleavage is another independent indicator of cell apoptosis. In Fig 6B and 6C and MIN6-HSD1 cells, we demonstrated a 2.2-fold increase in caspase-3 cleavage after 72 h treatment with DHC, compared to a 3-fold increase with DEX (Fig 6B, 2^nd^ and 4^th^ vs. 1^st^ bars). The effect of DHC was again blunted by the incubation with the 11β-HSD1 inhibitor (3^rd^ vs. 2^nd^ bars), reconfirming DNA fragmentation result in Fig 6A. In Fig 6C, using immunofluorescence, we further confirmed significantly increased caspase-3 cleavage in MIN6-HSD1 cells after being treated with DHC (middle panels, green spots highlighted by yellow arrows) which was largely abolished by the treatment with the 11β-HSD1 inhibitor (bottom panels). Our results in Figs 5 and 6 support the notion that intracrine activation of DHC by the action of 11β-HSD1 not only inhibits cell proliferation, but also causes significant apoptotic cell death.

## Discussion

Following our recent report, and from the same screening system [5], this study characterized yet another target of IGF-I action in pancreatic islets. We demonstrate that 11β-HSD1 is normally expressed in pancreatic islets, more specifically in the α-cells. Furthermore, IGF-I overexpression decreased the protein level and enzymatic activity, and direct treatment of IGF-I in isolated islets caused a late onset reduction of the protein level. The decrease in 11β-HSD1 in the islets of MT-IGF mice has functional consequences, as it was associated with a blunted response to the substrate of the enzyme (DHC) which normally inhibits insulin secretion in wild-type islets. To directly assess the function of this enzyme in MIN6 cells, 11β-HSD1 overexpression decreased cell proliferation and caused cell apoptosis in the presence of DHC. Thus, the activity of 11β-HSD1 and its inhibition by IGF-I seems to affect β-cell survival, proliferation and insulin secretion. Together with established roles played in liver and adipose tissues, the activity of 11β-HSD1 in pancreatic islets constitutes a putative, novel target of diabetic intervention.

Excess glucocorticoids lead to obesity, insulin resistance and even T2D, by promoting hepatic gluconeogenesis, central obesity and β-cell apoptosis [6, 25]. In the islet β-cells, glucocorticoids decrease Glut2 level, calcium influx and cellular cAMP generation by increasing α2-adrenergic receptors, all of which contribute to a general inhibition of insulin secretion [27–29]. In rodents, the active hormone corticosterone is predominantly bound to corticosteroid binding globulin, which is highly abundant and limits the hormonal effects [30]. In contrast, the substrate DHC is freely available in circulation and is readily activated in local tissues through the action of 11β-HSD1. Gene targeting experiments have clearly demonstrated that intracellular generation of corticosterone from DHC in the liver and adipose tissues constitutes a major component of glucocorticoid activities. 11β-HSD1 knockout causes a deficiency in DHC reductase, hyperandrogenism, precocious puberty and obesity in mice [8, 30]. These animals exhibit significant hypoglycemia upon starvation and after diet-induced obesity, supporting a physiological role of 11β-HSD1 in maintaining glucose production and/or inhibiting insulin secretion [30]. Our finding of islet-specific 11β-HSD1 expression is consistent to the overall model of intracrine secretion and that the expression and activity of 11β-HSD1 are both elevated in diabetic islets [9]. Moreover, DEX, DHC and corticosterone all inhibit insulin secretion, while the effect of DHC is abolished by 11β-HSD1 inhibitors [6, 17, 31]. In obese diabetic KKAy mice, diterpene isosteviol improves insulin sensitivity by decreasing 11β-HSD1 expression in pancreatic islets [32].

Early 11β-HSD1 was detected in the pancreatic islets of humans and ob/ob mice using RT-PCR [6, 31] and in an extensive acinar cell staining using immunohistochemistry [33]. Moreover, 11β-HSD1 was expressed specifically in the α- and PP-cells [17]. When mouse β-cells were purified from non-β cells, the expression level of 11β-HSD1 was greatly depleted, all supporting a non-β cell expression [22]. Consequently, our observation of islet α-cell expression was based on the use of two specific antibodies from Abcam and Santa Cruz. However, a recent report indicated its expression in human and rat β-cells based on immunohistochemistry, though we can only detect a faint, non-specific staining in some mouse islets using the same antibody (Abcam ab39364; data not shown) [18, 34]. Another report using a polyclonal sheep antibody provided by Dr. Scott Webster (University of Edinburgh) also indicated 11β-HSD1 staining throughout the islets [23, 35], both of which we have criticized and need to be reconciled by using independent techniques such as *in situ* hybridization.

In MT-IGF mice, ectopically overexpressed IGF-I could influence the α-cells and the expression of 11β-HSD1 through intra-islet paracrine or increased endocrine action [4]. We propose that corticosterone produced in the α-cells by the action of 11β-HSD1 could either inhibit insulin release directly via paracrine, or indirectly by inhibiting the secretion of glucagon which stimulates insulin release [17]. This would be consistent with 11β-HSD1-/- mice showing increased insulin but decreased glucose levels [30], and with the emerging importance of glucagon and α-cells in the etiology of diabetes mellitus [36]. Unexpectedly, a moderate, β-cell-specific overexpression of 11β-HSD1 was reported to boost islet compensation against high-fat diet through islet neogenesis and diminished inflammation [23]. We have reservations in accepting the “U-shaped” dose response because it directly challenged the diabetogenic role of glucocorticoids, and there was insufficient evidence to establish β-cell failure [35].

IGF-I inhibits 11β-HSD1 expression and its activity in adipocytes, stromal cells and hepatocytes [21, 24, 37, 38]. Conversely, glucocorticoids attenuate IGF-I action [39]. Through a whole genome screening, we first discovered a significant increase in 11β-HSD1 mRNA in the islets of MT-IGF mice [5], consequently decreased 11β-HSD1 protein level (albeit after 48 h) and enzyme activity *in vivo* in this study. The differential changes in mRNA and protein/activity levels may not be unreasonable as it has been found in yeast that for genes with equal mRNA levels, protein levels varied by more than 20-fold. For proteins with equal abundance, mRNA levels could vary by as much as 30-fold [40]. A recent report highlighted the notion that protein abundance is predominantly regulated at the ribosome by translational control [41]. In cycloheximide treated cells, IGF-I treatment resulted in the disappearance of 11β-HSD1 protein which indicates the possibility of IGF-I stimulated degradation. The controversy could further be caused by the restricted expression of 11β-HSD1 in islet α-cells, which has been supported by Fig 2 as well as two earlier reports [17, 22]. The real changes in 11β-HSD1 mRNA and protein could have been masked at least in part by an overwhelming majority of the transcripts/proteins contributed by the β-cells.

Glucocorticoids counteract IGF-I actions on islet β-cells in Akt phosphorylation, nuclear exclusion of FoxO1 and PDX1 activation, and resulted in cell death and diminished proliferation [42]. Our findings that IGF-I decreases 11β-HSD1 activity in the α-cells would help explain why MT-IGF islets have elevated basal insulin secretion *in vitro* (Fig 4A) and how islet β-cells might be protected from streptozotocin-induced apoptosis [4]. Regardless of the exact mechanism, our observation is interesting because IGF-I has not been known to regulate its level or activity in the pancreatic islets. Various 11β-HSD1 inhibitors decrease hepatic glucose production and fat mass, and are potential therapies against insulin resistance, obesity and T2D.

To further verify its functional relevance in islet β-cells, we evaluated possible changes in GSIS and the role of IGF-I and 11β-HSD1. As it has been well known, DEX inhibits GSIS from isolated islets of wild-type mice, and a similar effect was seen using DHC (Fig 4). In isolated islets of MT-IGF mice, DEX caused a significant reduction in glucose-stimulated insulin release, while the same dose of DHC was not as effective, indicating the consequence of diminished 11β-HSD1 activity. Two earlier studies reported that insulin secretion was suppressed by 11β-HSD1-mediated intracrine production of corticosterone [17, 31]. Incubation of the β-cells with DHC led to a dose-dependent inhibition of insulin release, which was reversed by the 11β-HSD1 inhibitor carbenoxolone [6]. The ability of DHC to reduce both the early and late phases of GSIS relies on 11β-HSD1 activity and the glucocorticoid receptor. Either elevated circulating glucocorticoids or increased intra-islet 11β-HSD1 or both, may increase the intracellular level of glucocorticoids and lead to inhibition of GSIS [31]. As 11β-HSD1 activity regulates insulin secretion and IGF-I decreases 11β-HSD1 activity, we believe IGF-I regulates insulin secretion at least in part by inhibiting 11β-HSD1.

To directly define the role of 11β-HSD1 on pancreatic islets, we stably overexpressed its cDNA in MIN6 cells and observed a decreased cell proliferation in the presence of DHC, as measured by the MTT assay and BrdU incorporation. The effect seemed to involve an inhibition of Akt phosphorylation (data not shown). In the meantime, the overexpression of 11β-HSD1 in the presence of DHC also triggered apoptosis of MIN6 cells as measured by caspase-3 activation and DNA fragmentation. The detrimental effects of 11β-HSD1 overexpression on both cell proliferation and apoptosis were reversed by a specific inhibitor of the enzyme.

In summary, our results support islet α-cell-specific expression of 11β-HSD1, and demonstrated an inhibitory effect of IGF-I on its expression and activity within the pancreatic islets, which may partially explain the decreased insulin secretion and/or previously reported islet protective effects of overexpressed IGF-I. Increased expression of 11β-HSD1 in mouse insulinoma cells render DHC-induced apoptosis and inhibition of cell proliferation. The inhibition of 11β-HSD1 activity by IGF-I in MT-IGF mice seems to exert a positive effect on pancreatic islets through islet preservation.

## Supporting Information

S1 Fig (DOCX)

S1 Table (DOCX)

S2 Table (DOCX)
