# Supplementary material for: Decreased 11β-Hydroxysteroid Dehydrogenase 1 Level and Activity in Murine Pancreatic Islets Caused by Insulin-Like Growth Factor I Overexpression
Source: PLoS One. 2015 Aug 25;10(8):e0136656. doi: 10.1371/journal.pone.0136656 (PMC4549276; doi:10.1371/journal.pone.0136656)
Supplement: S1 Fig — (DOCX) [file pone.0136656.s001.docx]

**S1 Figure.** The effect of acute IGF-I treatment (10^-7^ M) on 11β-HSD1 mRNA level in isolated pancreatic islets from C57BL/6 mice, N=4, *P<0.05 vs. untreated.
