# Supplementary material for: Decreased 11β-Hydroxysteroid Dehydrogenase 1 Level and Activity in Murine Pancreatic Islets Caused by Insulin-Like Growth Factor I Overexpression
Source: PLoS One. 2015 Aug 25;10(8):e0136656. doi: 10.1371/journal.pone.0136656 (PMC4549276; doi:10.1371/journal.pone.0136656)
Supplement: S1 Table — (DOCX) [file pone.0136656.s002.docx]

**S1 Table. Antibodies used in this study.**

| **Antigen** | **Dilution & Application** | **Host** | **Cat# Company** |
| --- | --- | --- | --- |
| 11β-HSD1 | 1:250-Western Blot, 1:100-Immunohistochemistry | Rabbit polyclonal | H-10 sc-20175, Santa Cruz |
|  | 1:500-Western Blot, 1:200- Immunohistochemistry | Rabbit polyclonal | ab83522, Abcam |
| HA Tag | 1:250 - Western Blot | Mouse monoclonal | G036, Abm |
| Glucagon | 1:100 - Immunohistochemistry | Goat polyclonal | C-18 sc-7779, Santa Cruz |
| Insulin | 1:100 - Immunohistochemistry | Guinea Pig polyclonal | ab7842, Abcam |
| β-actin | 1:3000- Western Blot | Mouse monoclonal | MM-0164-P, Medimabs |
|  | 1:5000- Western Blot | Rabbit, Polyclonal | Sc-1616-R, Santa Cruz |
| Cleaved caspase 3 | 1:1000-Western Blot  1:100-Immunohistochemistry | Rabbit polyclonal | 9661, Cell Signaling |
| Total caspase 3 | 1:1000-Western Blot  1:100-Immunohistochemistry | Rabbit polyclonal | 9662, Cell Signaling |
