# Supplementary material for: Decreased 11β-Hydroxysteroid Dehydrogenase 1 Level and Activity in Murine Pancreatic Islets Caused by Insulin-Like Growth Factor I Overexpression
Source: PLoS One. 2015 Aug 25;10(8):e0136656. doi: 10.1371/journal.pone.0136656 (PMC4549276; doi:10.1371/journal.pone.0136656)
Supplement: S2 Table — (DOCX) [file pone.0136656.s003.docx]

**S2 Table. 11β-HSD1 is most likely expressed by non-β cells: fold depletion in purified β-cells vs. total islets for representative genes.** Islets were isolated from MIP-GFP transgenic mice, GFP-positive β-cells were purified by flow cytometry. Transcription profiles were determined using next-generation mRNA sequencing (mRNA-seq). The ratio of the islet Cuffdiff-determined FPKM value divided by the β-cell value for each gene is shown. Based on Table 1 and supplemental table 1 of the publication and personal communication with Dr. Michael German of University of California, San Francisco [22].

| **Gene name** | **Known source of production** | **Islet/β-cell fold depletion** |
| --- | --- | --- |
| Glucagon | Endocrine α-cell | 15.18 |
| α−amylase Amy2a2 | Exocrine acinar cell | 28.4 |
| Gata2 transcription factor | Neuronal | 157.09 |
| Tcf21 tumor suppressor | Endothelial | 31.62 |
| 11β-HSD1 |  | 11.67 |
